# Advancing digital equity: A qualitative case study using the Implementation Research Logic Model

**DOI:** 10.1093/geroni/igaf122.2394

**Published:** 2025-12-31

**Authors:** Josie Santilli, Skye Leedahl, Jill Juris, Erica Liebermann, Casey McGregor

**Affiliations:** University of Rhode Island, Kingston, Rhode Island, United States; University of Rhode Island, Kingston, Rhode Island, United States; Appalachian State University, Boone, North Carolina, United States; University of Rhode Island, Kingston, Rhode Island, United States; University of Rhode Island, Kingston, Rhode Island, United States

## Abstract

Older adults without digital access, who are attempting to manage their lives within a digital landscape often struggle to maintain their independence. Technology is transforming the world around them, but they are excluded from participating. The purpose of the research was to explore how an evidence-based, university-led program for older adults, the iPad Project, was implemented to empower its participants with digital literacy. This qualitative case study used semi-structured interviews with 30 study participants and collaborators, a document review, and an Implementation Research Logic Model (IRLM) to investigate the interaction of elements, determinants, strategies, and mechanisms that impacted the outcomes. Interview questions were derived from relevant constructs in the Consolidated Framework for Implementation Research and the Expert Recommendation for Implementing Change strategies to explore the perspectives of project participants and collaborators. A thematic analysis of the responses was completed and informed the development of the IRLM which displayed how the project was implemented. The IRLM presented the key core and adaptable elements of the iPad Project that contributed to the project’s outcomes. Environmental determinants were found to impact these elements by challenging or facilitating project progress. Strategic approaches used to address these changing effects were identified and reflected how the project was managed. The interplay between hypothesized mechanisms and contextual determinants was examined and revealed the underlying influences that shaped the outcomes. The implementation outcomes were highlighted and illustrated the effect of this evidence-based intervention. This study provides guidance for future digital literacy program implementations.

